# Supplementary material for: Hexacyano Ferrate (III) Reduction by Electron Transfer Induced by Plasmonic Catalysis on Gold Nanoparticles
Source: Materials (Basel). 2019 Sep 17;12(18):3012. doi: 10.3390/ma12183012 (PMC6766293; doi:10.3390/ma12183012)
Supplement: Supplementary file 1 [file materials-12-03012-s001.pdf]

materials

MDPI

Article

Hexacyano Ferrate (III) Reduction by Electron Transfer Induced by Plasmonic Catalysis on Gold Nanoparticles

Iyad Sarhid [1] Isabelle Lampre [1,*] Diana Dragoe [2] Patricia Beaunier [3] Bruno Palpant [4] and Hynd Remita [1,5,*]

1 Laboratoire de Chimie Physique, Université Paris-Sud, UMR 8000 CNRS, Université Paris-Saclay, 91405 Orsay, France
2 Institut de Chimie Moléculaire et des Matériaux, Université Paris-Sud, UMR 8182 CNRS, Université Paris-Saclay, 91405 Orsay, France
3 Sorbonne Université, CNRS, Laboratoire de Réactivité de Surface, UMR 7197, F-75005 Paris Cedex 05, France
4 Laboratoire de Photonique Quantique et Moléculaire, UMR 8537 CentraleSupélec/Ecole Normale Supérieure Paris-Saclay/CNRS, Université Paris Saclay, 91190 Gif-sur-Yvette, France
5 CNRS, Laboratoire de Chimie Physique, UMR 8000 Université Paris-Sud, Université Paris-Saclay, 91405 Orsay, France
* Correspondence: Isabelle.lampre@u-psud.fr (I.L.); hynd.remita@u-psud.fr (H.R.)

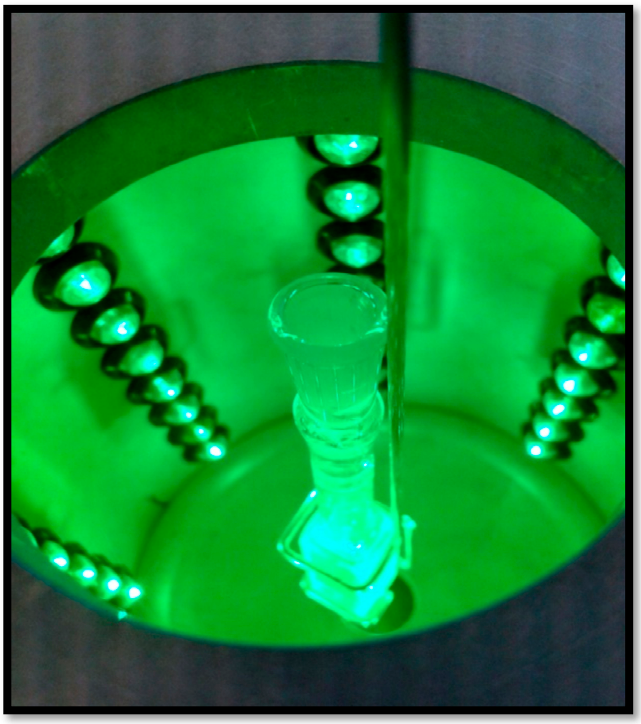

Figure S1. Photograph of the set-up used for light irradiation of the samples; homemade cylindrical photo-reactor equipped with 525 nm LEDs (Light Emitting Diods).

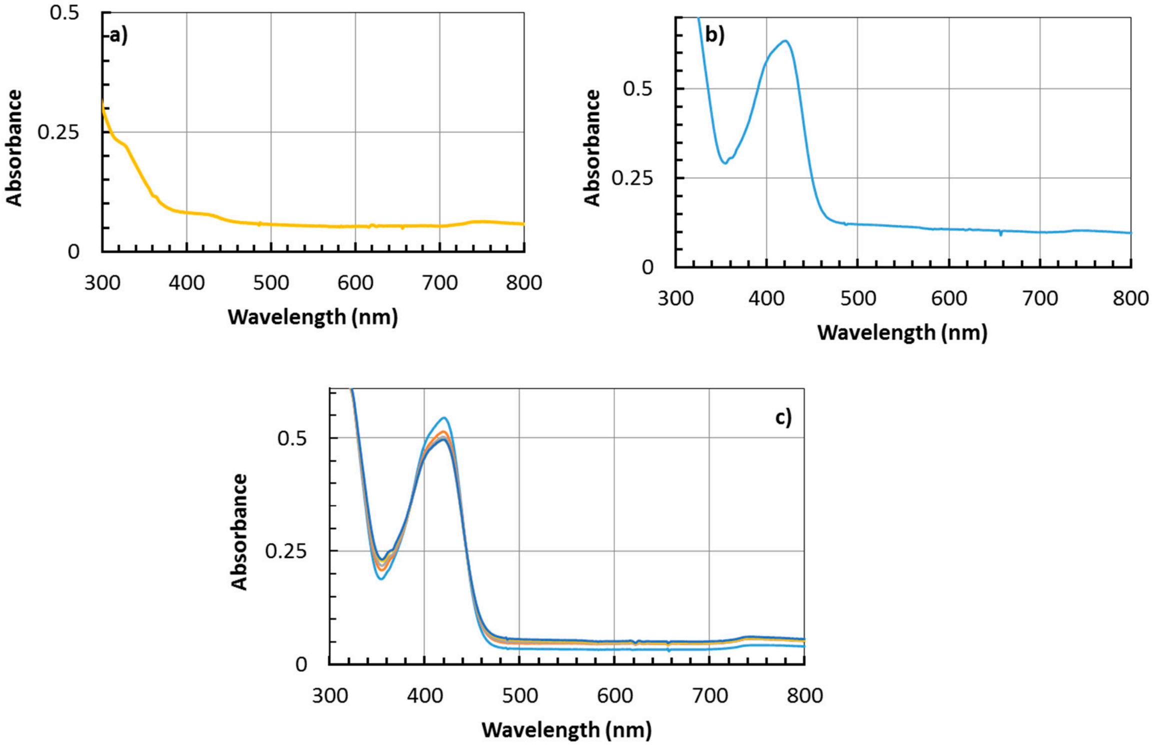

Figure S2. Absorbance spectra of (**a**) Sodium Thiosulfate 10^{-3} M; (**b**) Hexacyanoferrate 10^{-2} M (**c**) Mixture of both reagents under illumination: 20 mL of distilled water under visible irradiation for 120 min under N_2 (black spectrum before irradiation and blue spectrum after 120 min irradiation). The spectra were recorded using 2 mm quartz cell.

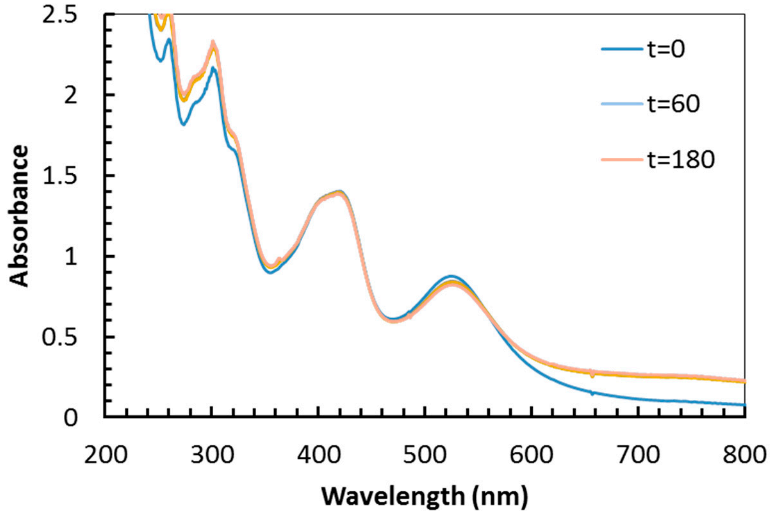

Figure S3. Temporal evolution of the absorption spectra of a solution containing 15 nm Au-NPs@citrate and 10^{-3} M potassium hexacyanoferrate III with no irradiation (in dark) for 180 min.

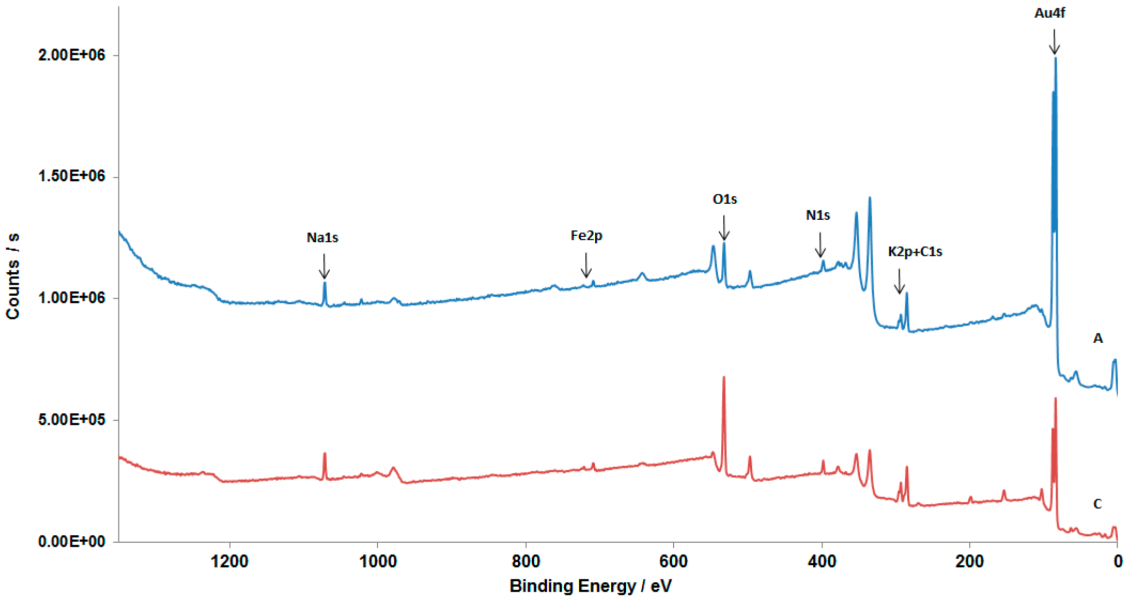

Figure S4. XPS wide-scan spectra of 30 nm Au-NPs@citrate after reaction under LEDs irradiation at 520 nm for deaerated solutions containing HC-FeIII with ST or without ST.

© 2019 by the authors. Licensee MDPI, Basel, Switzerland. This article is an open access article distributed under the terms and conditions of the Creative Commons Attribution (CC BY) license (http://creativecommons.org/licenses/by/4.0/).
